# Endoscopic Balloon Dilatation of Benign Esophageal Stricture—A Nonhazardous Procedure?

**DOI:** 10.1155/DTE.1.93

**Published:** 1994

**Authors:** Olle Ekberg, Anders Borgström, Frans-Thomas Fork, Eje Lövdahl

**Affiliations:** 1 The Department of Diagnostic Radiology University of Lund Malmö General Hospital Malmö S-214 01 Sweden; 2 The Department of Surgery University of Lund Malmö General Hospital Malmö S-214 01 Sweden; 3 The Endoscopy Unit University of Lund Malmö General Hospital Malmö S-214 01 Sweden

## Abstract

Balloon dilatation of benign esophageal strictures has been widely used since its introduction. We have performed 224 dilatation procedures in 52 patients. Dilatation was done as an outpatient procedure. Strictures were due to reflux esophagitis in 25 patients, anastomatic stenosis in 6, achalasia in 5, complications of sclerotherapy in 5, corrosive lesions in 3, and long-standing nasogastric intubation in 2. The cause was unknown in 6 cases. The intention was to dilate all strictures up to 20 mm. Three major complications occurred, and one of these patients died. The risk of perforation seems to be higher after repeated procedures than during the first one.


*Diagnostic and Therapeutic Endoscopy*, Vol. 1, pp. 93–97
Reprints available directly from the publisher
Photocopying permitted by license only

© 1994 Harwood Academic Publishers GmbH
Printed in Malaysia

### Endoscopic Balloon Dilatation of Benign Esophageal Stricture—A Nonhazardous Procedure?

OLLE EKBERG[1], ANDERS BORGSTRÖM[2], FRANS-THOMAS FORK[1], and EJE LÖVDAHL[3]

[1]*The Department of Diagnostic Radiology,* [2]*The Department of Surgery and* [3]*The Endoscopy Unit, University of Lund, Malmö General Hospital, S-214 01 Malmö, Sweden*

*(Received January 24, 1993)*

Balloon dilatation of benign esophageal strictures has been widely used since its introduction. We have performed 224 dilatation procedures in 52 patients. Dilatation was done as an outpatient procedure. Strictures were due to reflux esophagitis in 25 patients, anastomatic stenosis in 6, achalasia in 5, complications of sclerotherapy in 5, corrosive lesions in 3, and long-standing nasogastric intubation in 2. The cause was unknown in 6 cases. The intention was to dilate all strictures up to 20 mm. Three major complications occurred, and one of these patients died. The risk of perforation seems to be higher after repeated procedures than during the first one.

**KEY WORDS:** balloon dilatation; endoscopy, digestive system; esophageal perforation; esophageal stenosis; esophagus/radiography

### INTRODUCTION

Balloon dilatation of benign strictures of the esophagus has been widely accepted due to its advantage over other techniques. The radial direction of the force exerted by the inflated balloon on the stricture is considered to be the reason for the beneficial quality of this technique compared to other endoluminal procedures for dilatation (Kollath *et al.*, 1984). Balloon dilatation has also been recommended in cases inaccessible to other methods, be it narrow, long, or irregular strictures (Lindor *et al.*, 1985). Immediate relief of obstructive symptoms is reported in up to 98%, decreasing to around 60% after 3 months. The measurable, objective improvement is less, being 63 and 39%, respectively (Kozarek, 1986). The median duration of improvement is 12 months with a further procedure necessary in one out of three cases (Lindor *et al.*, 1985). The high degree of therapeutic safety is achieved by continuous, direct inspection and fluoroscopy during manipulation of floppy guidewires, flexible, soft catheters and the degree of balloon expansion during installation of contrast medium (Kollath *et al.*, 1984). Although the endoscopist has become more expert at handling strictures and thereby decreasing the necessity for surgical intervention (Earlam and Cuna-Melo, 1981), perforation is reported in 1 out of 100 (Götberg *et al.*, 1982), to 1 out of 300 cases (Kozarek, 1986). Minor complications given in the literature amount to 3% (Lindor *et al.*, 1985). Consensus has not been reached either as to optimal sizes of balloons, degree of pressure, and intervals between dilatations, or concerning the methods of evaluation of results. The aim of this study is to report our complication rate during and immediately after balloon dilatation.

### MATERIALS AND METHODS

The dilatation technique has been previously described (McLean *et al.*, 1987). It was preceded by an endoscopic inspection including biopsies in order to establish the benign nature of the stricture. The patients were routinely premedicated with 1.5 to 5 mg of Midazolam intravenously. A guidewire was inserted through the biopsy channel and advanced under fluoroscopic control well beyond the stenosis. The endoscope was removed, and an 8-cm long, desufflated 20 mm balloon was placed over the stricture with its midportion over the lesion. The balloon was filled with diluted contrast media until the hour-glass deformity disappeared.

The dilatation procedures used in this series involved the restoration of the esophageal lumen to 20 mm across.

Address for correspondence: Olle Ekberg, M.D., Department of Diagnostic Radiology, University of Lund, Malmö General Hospital, S-214 01 MALMÖ, Sweden

In only a few cases with long and hard strictures was the dilatation performed stepwise within a couple of days until a 20 mm lumen was achieved. In all patients, the efficacy of the dilatation was checked endoscopically and repeated within 1 month. Thereafter, the patients were instructed to visit the endoscopic unit spontaneously for further dilatation procedures when symptomatic. The degree of stenosis was given by the waist of the contrast-filled balloon and recorded in the report, but no esophageal barium examination was done routinely.

Charts of 52 consecutive patients, 31 men and 21 women, subjected to balloon dilatation from 1988 through 1991 were retrospectively studied. The median age at admission was 71 years, range 28–90. The etiology of the strictures was reflux esophagitis in 25, anastomotic stenosis in 6, achalasia and sequelae after sclerotherapy in 5 each, 3 corrosive lesions in long-standing nasogastric intubation in 2 but was unknown in 6 cases.

### RESULTS

In the 52 patients, 224 dilatation procedures were undertaken. The strictures were dilated to a minimum width of 20 mm except in 5 patients, in whom dilatation could only establish a lumen of 10–14 mm. Balloon dilatation was successful in 45 patients (66%). It had to be supplemented with Eder-Puestow bougienage in 5 patients. In 2 patients with anastomotic strictures, surgery was performed. A single dilatation was performed in 16 patients (31%), two or three procedures in 24 (46%), and more than 3 in 12 patients (23%). One patient with a recurrent stricture was dilated 25 times. The interval between dilatations ranged from 1–8 months.

Minor mucosal bleeding was observed in most cases, either at esophagoscopy, or as clots of blood on the balloon following treatment. Major hemorrhage was not recorded.

Three perforations were encountered (1.3%) among the 224 dilatations. One patient treated as an outpatient died at home 3 days after her 12th dilatation. At autopsy, a perforation site and mediastinitis were found. In this particular case, neither the patient nor the endoscopist noticed anything unusual during or immediately after the dilatation. This gives a mortality rate per procedure of 0.4% and a per patient rate of 1.9%.

The second perforation occurred during the 6th dilatation. The patient developed a pulmonary abscess caused by a perforation that was diagnosed 7 days after the endoluminal treatment. The patient was treated surgically and recovered without complications. (Fig 1)

The third patient had undergone 10 dilatations and the perforation was obvious at once due to severe pain. The patient went directly to thoracotomy, raphi, and drainage. The patient survived and has thereafter received 2 more dilatation procedures without complications.

### DISCUSSION

Balloon dilatation is an established method in the treatment of esophageal stricture. Dilatation can be made both endoscopically and/or under fluoroscopic control. Overall success rates have been reported to be 67–98%, and complication rates have been 0–9% in prior studies (Tytgat, 1989; Mandelstam *et al.*, 1976; Wesdorp *et al.*, 1982; McLean and LeVeen, 1989; La Berg *et al.*, 1985).

Endoluminal dilatation of benign esophageal strictures is considered a safe method, provided correct instrumentation is performed (Tytgat, 1989). Direct visualization of the stricture and placement of the guidewire through the endoscope, together with fluoroscopic control of the balloon treatment, is ideal and greatly recommended as precautionary steps, although there are reports in the literature presenting very low figures of perforation after dilatation without such visual control (Mandelstam *et al.*, 1976; Wesdorp *et al.*, 1982). According to common experience, the minimum diameter of an esophageal stricture that should be achieved as a result of dilatation in order to accomplish relief of symptoms is 13 mm (Tytgat, 1989). There are three basic types of devices used for nonsurgical esophageal dilatation: 1) rubber bougies filled with mercury, 2) fixed-sized dilatator passed over a guidewire (i.e., Eder-Puestow), 3) balloon dilatator also passed over guidewires, with or without endoscopic guidance. We now almost exclusively use the latter technique due to its ease and safety.

Using a balloon during dilatation, esophageal rupture is virtually eliminated. There are reduced shearing forces for the balloon compared with bougies *in vivo,* and this increases the margin of safety (McLean and LeVeen, 1989). Balloon dilatation is done during intravenous sedation and/or analgesics and is basically an outpatient procedure. The patient is observed in the recovery area for approximately 4–6 hours and then discharged if there is no evidence of chest pain or dysphagia. A post-procedure barium swallow may be added, but is probably not mandatory. However, when major complications occur, *i.e.*, esophageal rupture, prompt and adequate treatment including thoracotomy may be necessary.

Minor complications during the procedure are bleeding and chest pain. There may also be respiratory depression from oversedation. Such minor complications can be handled with the patient in an outpatient setting. Major symptoms, however, can be insidious and therefore many prefer to regularly do a barium or Gastrografin swallow after dilatation (La Berge *et al.*, 1985; Mucci, 1991). Therefore, it is necessary to be sure that the patient understands instructions to return immediately when symptoms such as chest pain, shortness of breath, and dysphagia occur after dilatation. There does not seem to be any relation between symptoms during the dilatation, and whether or not a late rupture will occur, *i.e.*, it is not those patients who experienced severe pain during the dilatation who will present with late rupture. However, it has been shown that early and late esophageal ruptures do predominantly occur in patients who have undergone several dilatations. Many patients experience immediate symptom relief, *i.e.*, they can eat normally. However, the patient should be instructed to take liquids and only soft food during the day of the procedure and return to solid food the morning after. Some patients get reflux of gastric content to the dilated stenosis. Such reflux of acid material can give heartburn that can be difficult to distinguish from pain due to rupture. Many of these patients, however, are on $H_2$-blockers and therefore do not experience such heartburn.

We encountered no hemorrhage requiring treatment. However, three perforations were experienced, all occurring after several preceding successfully accomplished therapeutic interventions, *i.e.*, 6, 10, and 12 times, respectively. The perforation was immediately diagnosed and treated in only one of these cases. The other two cases revealed themselves hours to days later; one having developed a pulmonary abscess (Fig 1), while one patient was found dead at home 72 hours post-procedure. The vague and late symptoms of perforation is confusing and bring the question of patient monitoring to the fore. The vast majority of our patients are treated on an out-patient basis and are requested to remain at the endoscopic unit for 4–6 hours after dilatation. They are also requested to stay in touch and report development of any new symptoms or when deglutition has not improved. These precautions have apparently been inadequate, in spite of the fact that all of the patients were accustomed to the procedure and knew how to get in contact with the department in case of emergency. As a consequence, we are now actively calling the patients within 24 hours of treatment in order to find symptoms that might be compatible with an unsuccessful treatment or any procedure-related complication.

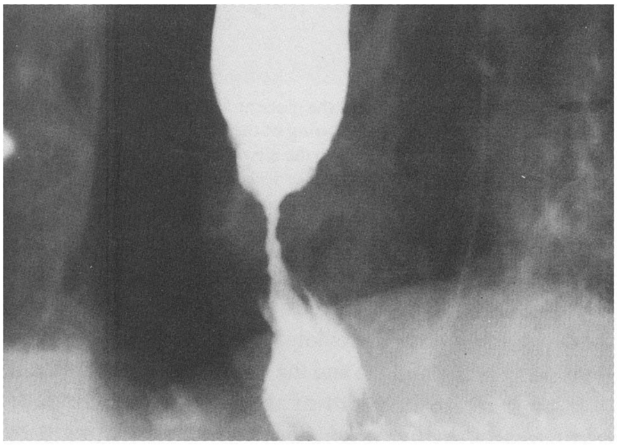

**Figure 1a** 53-year-old male with severe GERD. There is a stricture in the distal esophagus.

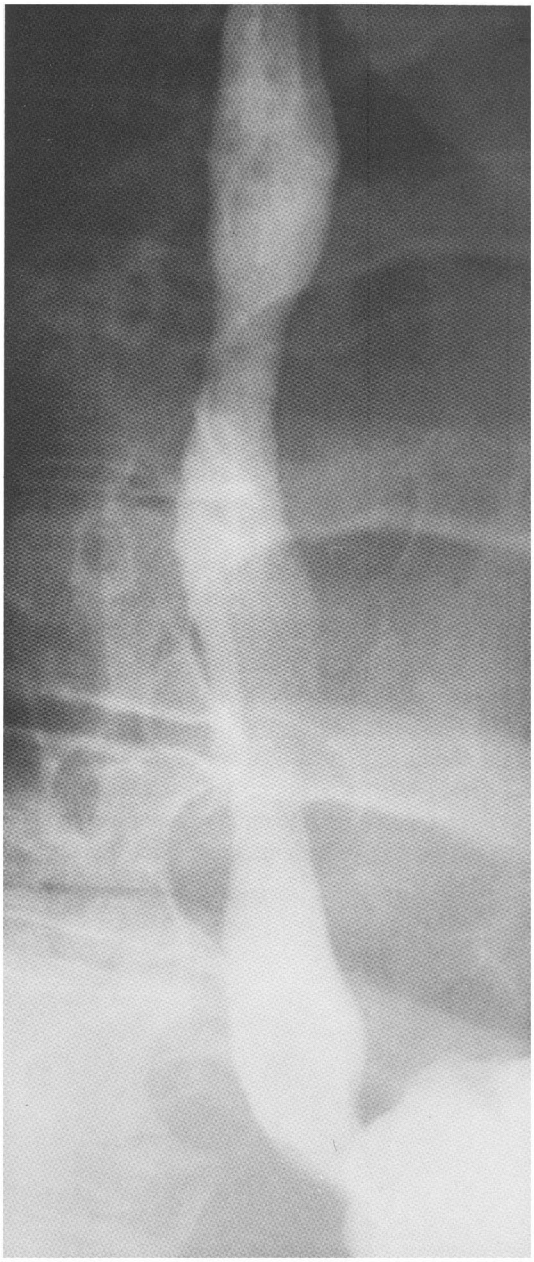

**Figure 1b** Balloon dilatation was uneventful and a post-procedure Gastrografin swallow showed patency of the esophageal lumen.

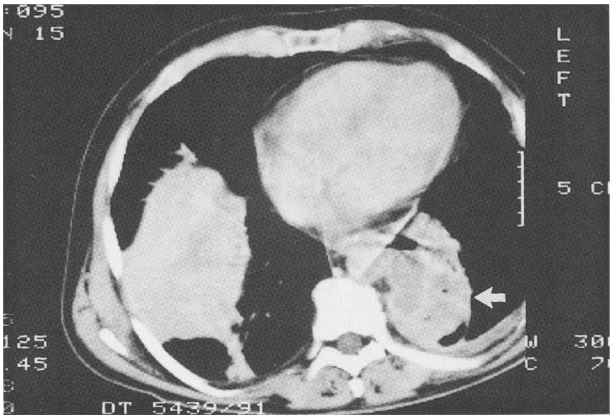

**Figure 1c** Forty-eight hours later, the patient was admitted to the hospital with chest pain, shortness of breath, and dysphagia. A CT examination of the lower thorax showed a large abscess (arrow) adjacent to the esophagus.

Our material can neither answer the question of the length of interval between procedures nor that of the number of therapeutic sessions needed to achieve persistent relief of symptoms. After the initial treatments, the patients were asked to spontaneously visit the endoscopy unit as soon as deglutition symptoms reoccurred.

Balloon dilatation treatment was insufficient in 7 of our 52 patients (13%). Two patients with anastomotic strictures had to be operated on due to rapid restenosis even after several dilatations. In five patients, the balloon dilatation had to

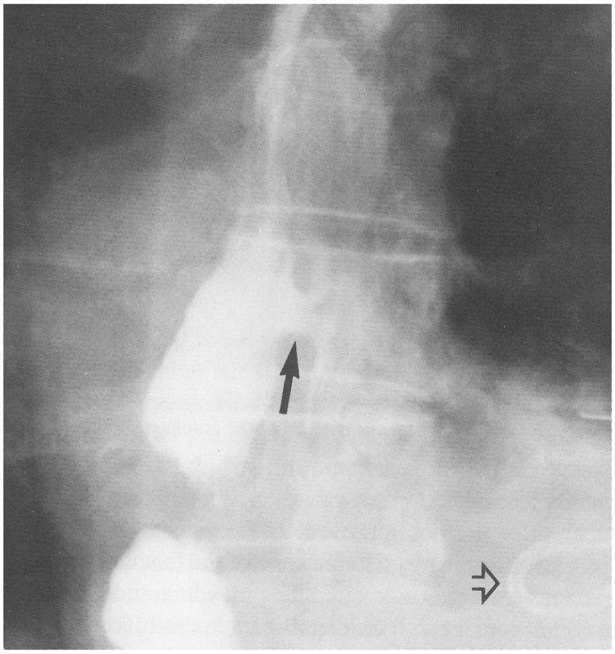

**Figure 1d** A new Gastrografin study showed a leak (arrow) from the proximal area of the dilated stricture and the left thoracic cavity. A percutaneous drainage catheter (open arrow) had been placed under ultrasound guidance.

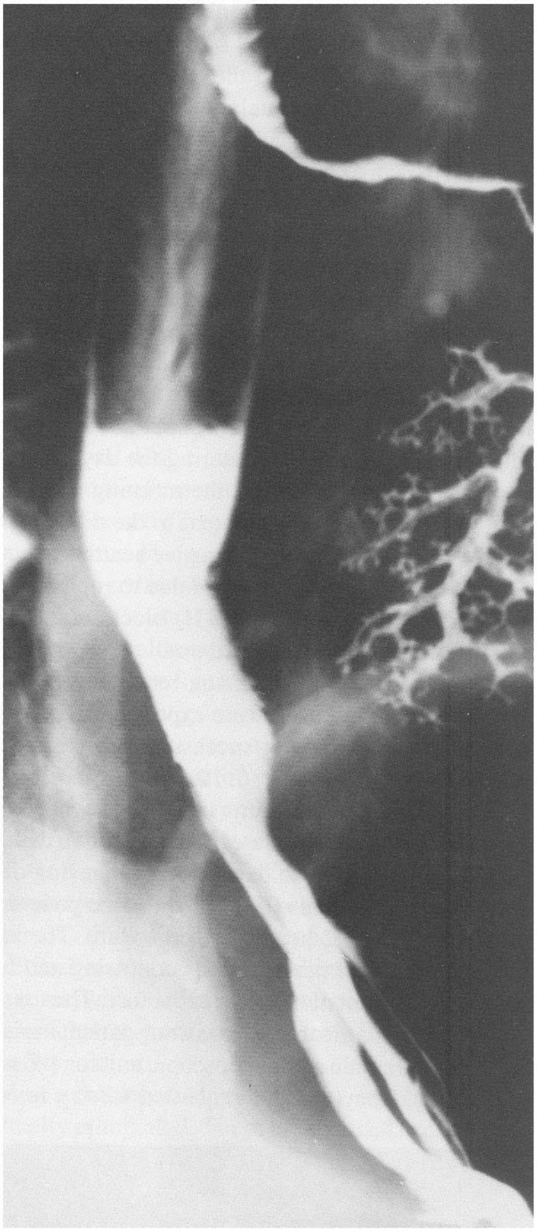

**Figure 1e** Six months later, the patient had recovered. A barium swallow showed only mild narrowing of the distal esophagus. There is, however, aspiration of barium into the airways. The latter was probably not related to the balloon dilatation or its complications.

be completed with occasional (one–seven) dilatations using the Eder-Puestow instrument. Interestingly enough, two of our three perforations occurred after pneumatic dilatation in those patients who had previously been subjected to dilatations using both balloons and the Eder-Puestow instrument.

If no increase in width of the stricture can be radiographically documented, another treatment might be contemplated, be it by using soft dilators (McLean *et al.*, 1987), or surgery. A barium-swallow will also reveal any concomitant esophageal motor disturbance in still symptomatic patients successfully dilated.

In conclusion, there is a risk of late perforations after balloon dilatation of benign strictures (0.4% per procedure and 1.9% per patient). Furthermore, the risk of perforations seems higher after repeated procedures than during the first dilatation. Dilated patients should be carefully monitored after the procedure. Anastomic stricture seems to be difficult to dilate as 2 of 6 (33%) had to undergo surgery for achievement of 20 mm diameter. In spite of this, we feel that balloon dilatation of peptic strictures of the esophagus is an outpatient procedure that can be safely done in a cooperative patient who has been carefully instructed about possible late complications and who can reach his physician easily during the next few days after the procedure.

### REFERENCES

Dawson S. L., Mueller P. R., Ferrucci J. T., *et al.* (1984) Severe esophageal strictures: indications for balloon catheter dilatation. *Radiology*, 153:631–635.

de Lange E. E., Shaffer H. A. (1988) Anastomotic strictures of the upper gastrointestinal tract: Results of balloon dilatation. *Radiology*, 167:45–50.

Earlam R. Cuna-Melo J. R. (1981) Benign oesophageal strictures: historical and technical aspects of dilatation. *Br J Surg*, 68:829–836.

Götberg S., Afzelius L.-E., Hambraeus G., *et al.* (1982) Balloon-catheter dilatation of strictures in the upper digestive tract. *Radiology*, 22:479–483.

Kollath J., Starch E., Vittorio P. (1984) Dilatation of esophageal stenosis by balloon catheter. *Cardiovasc Intervent Radiol*, 7:35–39.

Kozarek R. A. (1986) Hydrostatic balloon dilation of gastrointestinal stenoses: a national survey. *Gastrointest Endosc*, 32:15–19.

La Berge J. M., Kerlan R. K., Pogany A. C., Ring E. J. (1985) Esophageal rupture, complication of balloon dilatation. *Radiology*, 157:56.

Lindor K. D., Ott B. J., Hughes R. W, Jr. (1985) Balloon dilatation of upper digestive tract strictures. *Gastroenterol*, 89:545–548.

McLean G. K., Cooper G. S., Hartz W. H., *et al.* (1987) Radiologically guided balloon dilatation of gastrointestinal strictures. Part I. Technique and factors influencing procedural success. *Radiology*, 165:35–40.

McLean G. K., LeVeen R. F. (1989) Sheer stress in the performance of esophageal dilatation: comparison of balloon dilatation and bougienage. *Radiology*, 172:983–986.

Mandelstam P., Sugawa C., Silvis S. E. (1976) Complications associated with esophagogastroduodenoscopy and with esophageal dilatation. *Gastrointest Endosc*, 23:16.

Maynar M., Guerra C., Reyes, R. *et al.* (1988) Esophageal strictures, balloon dilatation. *Radiology*, 167:703–706.

Mucci B. (1991) Oesophageal ruptures complicating balloon dilatation of strictures: a report of two cases. *Br J Radiol*, 64:1060–1061.

Starck E., Paolucci V., Herzer M., Crummy A. B. (1984) Esophageal stenosis treatment with balloon catheters. *Radiology*, 153:637–640.

Tytgat G. N. J. (1989) Dilation therapy of benign esophageal stenoses. *World J Surg*, 13:142–148.

Wesdorp I. C. E., Huibregtse K., Tytgat G. N. (1982) Results of conservative treatment of benign esophageal strictures: a follow-up study in 100 patients. *Gastroenterology*, 82:487.

